# Anti-inflammatory effect of fullerene C_60_ in a mice model of atopic dermatitis

**DOI:** 10.1186/s12951-016-0159-z

**Published:** 2016-01-25

**Authors:** Nadezda Shershakova, Elena Baraboshkina, Sergey Andreev, Daria Purgina, Irina Struchkova, Oleg Kamyshnikov, Alexandra Nikonova, Musa Khaitov

**Affiliations:** NRC Institute of Immunology FMBA of Russia, Moscow, Russia

**Keywords:** Fullerene C_60_, Atopic dermatitis, Mouse model, Cytokine

## Abstract

**Background:**

Water-soluble form of fullerene C_60_ is a promising tool for the control of ROS-dependent inflammation including allergic diseases. Anti-inflammatory effects of C_60_ (nC_60_) aqueous dispersion were evaluated in the mouse models of atopic dermatitis using subcutaneous (SC) and epicutaneous (EC) applications during 50 days period. A highly stable nC_60_ was prepared by exhaustive dialysis of water-organic C_60_ solution against water, where the size and ζ-potential of fullerene nanoparticles are about 100 nm and −30 mV, respectively.

**Results:**

To induce skin inflammation, female BALB/c mice were EC sensitized with ovalbumin three times during one-weekly exposures. The nC_60_ solution was administrated in mice subcutaneously (SC) (0.1 mg/kg) and epicutaneously (EC) (1 mg/kg). Significant suppression of IgE and Th2 cytokines production and a concomitant rise in concentrations of Th1 cytokines were observed in nC_60_-treated groups. In addition, a significant increase in the levels of Foxp3^+^ and filaggrin mRNA expression was observed at EC application. Histological examination of skin samples indicated that therapeutic effect was achieved by both EC and SC treatment, but it was more effective with EC. Pronounced reduction of the eosinophil and leukocyte infiltration in treated skin samples was observed.

**Conclusions:**

We suppose that nC60 treatment shifts immune response from Th2 to Th1 and restores to some extent the function of the skin barrier. This approach can be a good alternative to the treatment of allergic and other inflammatory diseases.

## Background

Atopic dermatitis (AD) is a chronic inflammatory skin disease that predominantly affects children and is characterized by skin lesions, persistent erythema, scaling, excoriations, and pruritus. In addition, the disease is commonly associated with allergic rhinitis and asthma. The number of AD patients increased by 10–30 % in children and 2–10 % in adults in the last 30 years worldwide [1–3]. AD is the result of complex interactions among several genetic factors, deficiencies in skin barrier function, exposure to various allergens and infectious agents and features of the immune response [4–6]. In 60 % of patients, AD starts before the age of 6 years, in 18 % the onset is after the age of 20 [7]. Its pathogenesis involves impairments in the skin barrier, allowing abnormally enhanced dermal presentation of antigens/allergens to the immune system [8, 9]. In many AD patients Th2/IgE-mediated allergic reactions play leading role, however, Th2 cells predominate in the initial stage lesions with a switch to Th1-cells in a chronic phase. As a rule, diseases arising from a dysfunction of immune cells and/or their products often manifest with skin symptoms [10]. Many observations suggest that allergic and inflammatory skin diseases like AD are mediated by oxidative stress [11–13]. Mast cells generate mainly intracellular reactive oxygen species (ROS) following the aggregation of FceRI; these ROS may act as secondary messengers in the induction of several biological responses [14]. A generation of ROS induces oxidative protein damage in the stratum cornea, which leads to the disruption of barrier function and the exacerbation of AD [15, 16].

Fullerene is a molecular carbon form with cage spheroid structure, and it possesses strong antioxidant activity. At present, the fullerene C_60_ is produced industrially in large quantities and is commercially available. Its unique properties include strong electron-acceptor activity, high polarizability and hydrophobicity; a large surface of C_60_ molecule allows attachment of various hydrophilic addends, providing prospects for the design of new biomedical products. Fullerene and some of its derivatives as potent antioxidants (in vitro and in vivo) are capable of effective ROS inactivation [17]. An important feature of fullerene C_60_ is its low toxicity [18, 19], a permeability through biological membranes [20, 21], and lack of immunogenicity [22]. Thus, fullerene presents a promising therapeutic agent for the treatment of allergies and other inflammatory diseases such as Parkinson’s, Alzheimer’s and AD.

Anti-inflammatory activity of fullerene derivatives was recorded by several investigators, and, in particular, researchers from the Luna group (led by Dr. C. L. Kepley) were the pioneers in this field [23–26]. Some C60-drived compounds were able to inhibit an IgE-dependent allergic response. For instance, fullerenol (C_60_(OH)_n_) and amino-fullerene (C_60_(NHCH_2_CH_3_)_n_) inhibited in vitro IgE-dependent degranulation of mast cells and secretion of cytokines and prostaglandins in response to allergen and authors suggested that this effect is associated with lower levels of ROS in the stimulated cells [23]. On the other hand, the C_60_ aqueous dispersion obtained by Andrievsky’s method [27] induced gene expression of proinflammatory cytokines (IL-1, TNF-α, IL-6) and Th1 cytokines (IL-12, IFN-γ) in mice via aerosol administration to the airways [28]. Aqueous dispersion of C_60_ (nC_60_) provided inhibitory effect on IgE-mediated histamine release from peripheral blood basophils in vitro and suppressed anaphylaxis in mice caused by administration of ovalbumin [29]. Similar C_60_ dispersion exhibited significant modulator activity on the DTH reaction suppressing Th1 cytokines release [30, 31].

Fullerene C_60_ in aqueous media always exists in form of nanoparticles (clusters), and hence, the degree of their possible undesirable effect is still being discussed [32, 33]. In several studies, nanoparticles are perceived as toxic agents, in others—the damage is not detected. As was shown, the polystyrene nanoparticles aggravated AD-like skin lesions [34] and metal oxide nanoparticles such as ZnO induced systemic production of IgE antibodies [35]. Currently, nanoparticles were used for transcutaneous drugs delivery, but not for the AD therapy [36, 37]. It should be noted, that the use of fullerene at the AD has not been described.

The purpose of this study was to evaluate the therapeutic properties of the nC_60_ obtained by novel biocompatible method for the treatment of AD induced in a mouse model [38].

## Results

### Aqueous fullerene solution, nC_60_

Recently, we have proposed a new simple method for preparation of C_60_ aqueous solution using a “dialysis principle” [39]. Shortly, the protocol design includes the dissolution of crystalline fullerene in N-methylpyrrolidone (NMP), dilution of this solution with distilled water or aqueous solution of an l-amino acid used as a stabilizing agent with subsequent exhaustive dialysis against deionized water. Thus, the protocol excludes the use of toxic organic solvents (toluene, tetrahydrofuran) as well as sonication, heating and durable mixing frequently used in the known methods [27, 40, 41]. This approach provides a high conversation of the fullerene C_60_ from the crystalline state to the solution with concentration of C_60_ up to 1 g/l (at additional vacuum concentration) and hydrodynamic size of C_60_ particles about 80–100 nm (Table 1). Absorption spectrum of nC_60_ is characterized by three intense maxima at 219, 265 and 344 nm and weak broad band between 400 and 500 nm, and it practically did not differ from those of nC60 obtained through other methods. FTIR spectra of dried nC_60_ shows vibration bands of free C_60_ molecules at 1182 and 1428 cm^−1^ and additional ones at 3500–3200, 1650–1660, and 1000–1100 cm^−1^ due to obvious presence of residual water molecules and NMP (as a donor–acceptor complex). Calculations on the molar ratio of NMP to fullerene displayed the value 0.7–1.1, while a calculation of the ratio H_2_O to C_60_ based on the hydrogen content gave values from 5 to 10. The NMP is known to have low toxicity via oral, dermal and inhalation routes of delivery. In medicine, it has a long track recorded as a constituent in medical devices approved by the European Commission and FDA and thus can be considered as safe [42]. In addition, good heat resistance enables to sterilize the C_60_ solution prior to its administration into the body. In this study, concentration of C_60_ in the stock nC_60_ was 120 µg/ml.

**Table 1:** Physical parameters of the nC_60_

Parameter	Value
Feasible concentration^a^	~1 mg/ml
Average size (nm)	80–100 nm
PDI (polydispersity index)	0.175 ± 5
Zeta potential (mV)	–27/30
UV–Vis major absorption peaks (nm)	219 (s), 265 (s), 344 (s), 450 (bw)
FTIR spectra (cm^−1^)	525, 1000–1100, 1182, 1428, 1650–1660, 3200–3500
Mol. ratio C_60_:NMP	~1
Mol. ratio C_60_:H_2_O	5–10

*s* strong, *bw* broad weak

^a^With optional vacuum evaporation

### nC_60_ modulates allergen-specific antibody production

**Fig. 1:**
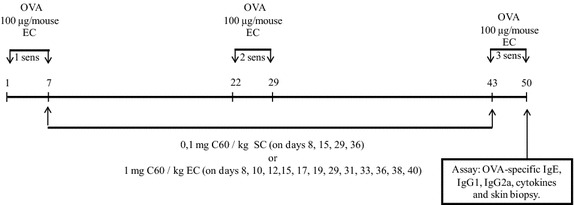
Sensitization protocol and nC_60_ treatment. Mice were sensitized with OVA (100 µg) or saline applied at 100 µl to a sterile patch. The patch was applied for 1 week and then removed. nC_60_ treatment was done between the applications (sens): SC (0,1 mg nC_60_/kg) on days 8, 15, 29, 36; EC (1 mg nC_60_/kg) on days 8, 10, 12, 15, 17, 19, 29, 31, 33, 36, 38, 40. The control group (“placebo”) had no any treating

**Fig. 2:**
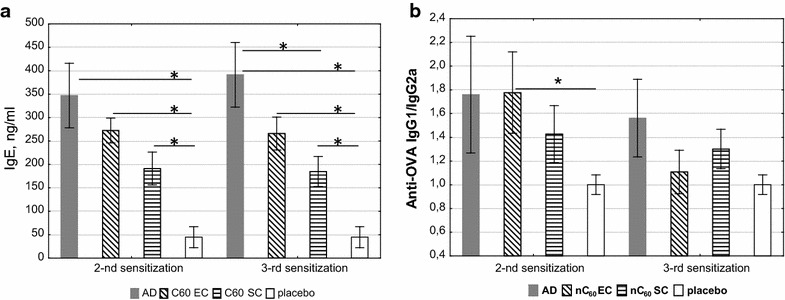
Changes in concentrations of OVA-specific antibodies in sera obtained after treatment with nC_60_ detected by ELISA. The results are presented as mean value (mean ± SE, n = 8 for each). AD, OVA-sensitized mice (AD model); nC_60_ EC, OVA-sensitized mice treated with nC_60_ by EC; nC_60_ SC, OVA-sensitized mice treated with nC_60_ by SC; placebo, PBS-sensitized mice. **a** OVA-specific IgE response, **b** OVA-specific IgG1/IgG2a ratio (*p < 0.05)

To induce a mouse AD [38, 43, 44], female BALB/c mice were EC sensitized by skin application with 100 μg doses of OVA in PBS (1 mg/ml) during three one-week exposures with two-week intervals as described in “Materials and methods” and showed in Fig. 1 (“AD” group). Mice received nC_60_ by both EC (“nC_60_ EC”) and SC (“nC_60_ SC”) administration routes in doses 1 mg/kg and 0.1 mg/kg of nC_60_, respectively, between OVA-applications. Control group received PBS-application only (“placebo”). Figure 2 shows that OVA-specific IgE and IgG1 levels in sera were elevated in OVA-sensitized mice (“AD” group). The main question was whether nC_60_ treatment could change OVA-specific antibody level in OVA-sensitized mice.

To assess the effect of nC_60_-treatment on specific IgE and IgG production sera were collected after 2-nd and 3-rd sensitizations. As can be seen from Fig. 2a, specific IgE concentrations in OVA-sensitized mice were reduced after nC_60_ treatment. SC administration of nC_60_ had stronger inhibition effect on IgE production compared with the EC one. This tendency was observed both after the 2-nd and 3-rd OVA-sensitizations (2 and 3 sens, Fig. 2). After the last sensitization the reduction of IgE concentration was about 50 % (statistically significant). OVA-specific IgG1 and IgG2a levels and IgG1/IgG2a ratio as markers for Th2 and Th1 responses [45] were measured for each group, and results (Fig. 2b) suggest that the nC_60_ treatment had no statistically significant effect on the specific antibody production (although a decreasing trend, especially after 3-rd sensitization was observed).

### Fullerene-induced cytokine profile alterations

**Fig. 3:**
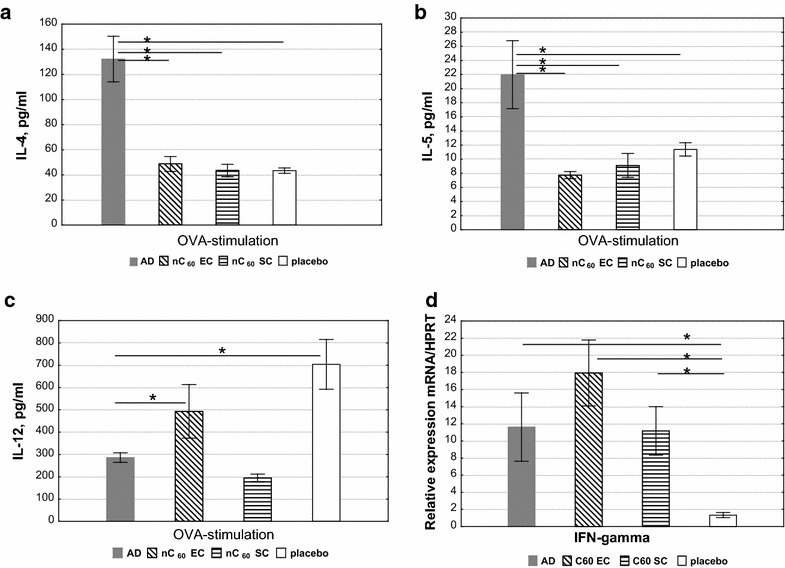
The levels of cytokines IL-4 (**a**), IL-5 (**b**) and IL-12 (**c**) in supernatants of OVA-stimulated mouse splenocytes taken after 3-rd sensitization and incubated with OVA for 72 h (quantified by ELISA). Relative expression of IFN-γ mRNA (**d**) in mouse splenocytes taken after 3-rd sensitization and incubated with OVA for 72 h (quantified by real-time PCR). The results are presented as a mean concentration (mean ± SE, n = 8 for each). Designations of *columns* in the diagram are the same as those in Fig. 2; *p < 0.05

Analysis of cytokine expression was carried out after the 3-rd sensitization in supernatants of OVA-stimulated mouse splenocytes (3 sensitization, Fig. 1). It was shown that the nC_60_ exerts a strong effect as the IL-4 concentration was decreased by 2.5 times compared with “AD” group regardless of the route of administration (Fig. 3a). The same effect we also observed for IL-5. In this case, a slightly stronger effect was observed via EC application (Fig. 3b).

Figure 3c shows that the IL-12 concentration was significantly higher in group “nC_60_ EC” and “placebo” compared with “AD” and “nC60 SC” group. IFN-γ expression was significantly increased in nC_60_ group compared with “AD” and normal-control (“placebo”) Fig. 3d. Moreover, the strongest effect was observed after EC/nC_60_ treatment.

### Foxp3 + regulatory cells (Foxp3 + Tregs) induction

**Fig. 4:**
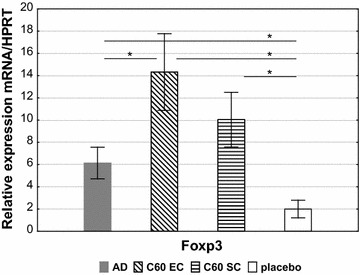
Foxp3 expression. The specific mRNA in OVA-stimulated mouse splenocytes (incubated with OVA for 72 h) were quantified by real-time PCR. The results are presented as mean mRNA expression (mean ± SE, n = 8 for each). The relative levels of Foxp3 expression were calculated by referring to the HPRT (hypoxanthine guanine phosphoribosyltransferase) in each sample. AD: AD mouse model; nC_60_ EC: OVA-sensitized mice treated with nC_60_ EC; nC_60_ SC: OVA-sensitized mice treated with nC_60_ SC; placebo: PBS-sensitized mice (*p < 0.05)

In this study, we have measured the Foxp3 expression in OVA-stimulated mouse splenocytes to evaluate the level of Foxp3 + Tregs after the nC_60_ treatment. As we can see a significant increase in Foxp3 expression was observed in the “nC60 EC” group compared with “AD” and “placebo” (Fig. 4). The Foxp3 level was only slightly higher in group “nC_60_ SC” than in “AD”.

### Analysis of filaggrin expression

**Fig. 5:**
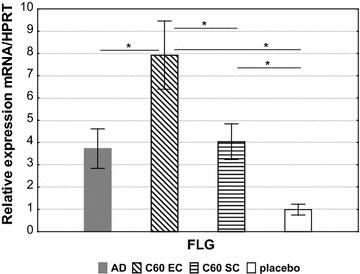
FLG expression. The specific mRNA in OVA-stimulated mouse splenocytes (incubated with OVA for 72 h) were quantified by real-time PCR. The results are presented as mean mRNA expression (mean ± SE, n = 8 for each). The relative levels of FLG expression were calculated by referring to the HPRT (hypoxanthine guanine phosphoribosyltransferase) in each sample. AD: AD mouse model; nC_60_ EC: OVA-sensitized mice treated with nC_60_ EC; nC_60_ SC: OVA-sensitized mice treated with nC_60_ SC (*p < 0.05)

We used a quantitative real-time PCR to evaluate the effect of nC_60_ on expression the FLG in AD-induced mice (Fig. 5). It was shown that the nC_60_ potently promoted the FLG expression. The most significant increase of FLG was observed at EC/nC_60_ application, in this case, FLG expression was increased about 3 times (p < 0.05).

### Histological assay

**Fig. 6:**
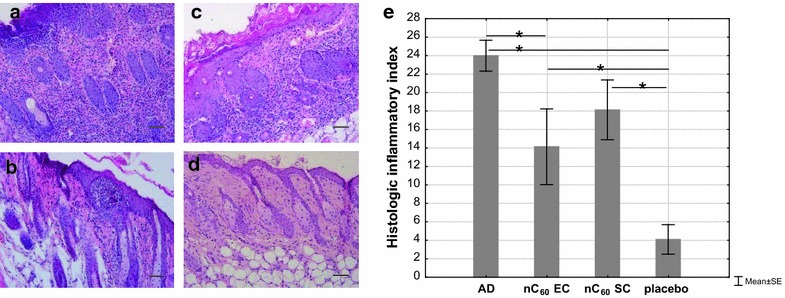
Histologic features (*scale bar* 100 μm). **a** Mice sensitized with OVA and treated with PBS (“AD”); **b** mice sensitized with OVA and treated with nC_60_ EC (“nC_60_ EC”); **c** mice sensitized with OVA and treated with nC_60_ SC (“C_60_ SC”); **d** mice sensitized with PBS (“placebo”); **e** evaluation of skin lesions by summary histological index given in Table 1

Skin biopsies from sensitized mice were taken after the final 3-rd sensitization. Skin sections were stained with H&E and examined at 100-fold magnification. The skin samples from the “AD” group had dermal and epidermis necrosis from severe to medium degree. There were visible marked epidermis hyperplasia, pronounced diffuse mixed leukocyte infiltrates with eosinophils prevalence both in dermis and epidermis (Fig. 6a). The cellular infiltrate consisted of neutrophils, eosinophils, and lymphocytes, while the group “placebo” did not present any pathologic changes showing almost normal appearance (Fig. 6d). The infiltration degree of leukocytes in dermis and epidermis were decreased in SC/nC_60_-treated mice (“nC_60_ SC”) compared with “AD”, however, the eosinophils amount was the same as in the “AD” (Fig. 6c). In this case, we observed decrease in the epidermis necrosis and destructive hemorrhage in derma. There was a moderate epidermal hyperkeratosis obviously as a compensatory protection mechanism against damage. Figure 6b shows that the most pronounced nC_60_ therapeutic effect was in the case of EC nC_60_-treated mice (“nC_60_ EC”). The leukocytes infiltration degree and the eosinophils number were significantly reduced in “nC_60_ EC” group compared with “AD” and “nC_60_ SC”. It was shown, that the epidermal necrosis, destructive hemorrhage in dermis and hyperkeratosis either were absent or were mild.

We evaluated the histologic pictures in semi-quantitative histological index (score) comparing nC_60_-treated and non-treated groups with each other based on the microscopic features. Epidermal thickening, epidermal necrosis, epidermal hyperkeratosis, dermal and subcutaneous fat necrosis, swelling, hemorrhage, and cell infiltration of dermis and subcutaneous fat were the main evaluation criteria for the histologic skin lesions (Table 2). Each parameter had a degree of manifestation, estimated notional value and appropriate score.

**Table 2:** The main assessment criteria for histological skin lesions

No.	Criterion	The degree of manifestation	Score
1	Epidermal thickening	Absent	0
		Mild	1
		Moderate	2
		Pronounced	3
2	Epidermal necrosis	Absent	0
		Present	1
3	Epidermal hyperkeratosis	Absent	0
		Present	1
4	Connective tissue like dermal proliferation	Absent	0
		Mild	1
		Moderate	2
		Pronounced	3
5	Dermal necrosis	Absent	0
		Present	1
6	Dermal swelling	Absent	0
		Present	1
7	Dermal hemorrhage	Absent	0
		Present	1
8	Connective tissue like subcutaneous fat proliferation	Absent	0
		Mild	1
		Moderate	2
		Pronounced	3
9	Subcutaneous fat necrosis	Absent	0
		Present	1
10	Subcutaneous fat swelling	Absent	0
		Present	1
11	Subcutaneous fat hemorrhage	Absent	0
		Present	1
12	Cell infiltration of dermis	Absent	0
		Mild	1
		Moderate	2
		Pronounced	3
13	Cell infiltration of dermis (eosinophils)	Absent	0
		Mild	1
		Moderate	2
		Pronounced	3
14	Cell infiltration of dermis (polynuclear leukocytes)	Absent	0
		Mild	1
		Moderate	2
		Pronounced	3
15	Cell infiltration of dermis (lymphocytes)	Absent	0
		Mild	1
		Moderate	2
		Pronounced	3
16	Cell infiltration of subcutaneous fat	Absent	0
		Mild	1
		Moderate	2
		Pronounced	3
17	Cell infiltration of subcutaneous fat (eosinophils)	Absent	0
		Mild	1
		Moderate	2
		Pronounced	3
18	Cell (polynuclear leukocytes) infiltration of subcutaneous fat	Absent	0
		Mild	1
		Moderate	2
		Pronounced	3
19	Cell (lymphocytes) infiltration of subcutaneous fat	Absent	0
		Mild	1
		Moderate	2
		Pronounced	3

Figure 6e shows that the maximum inflammatory index was observed in the “AD” (score = 24) while the “placebo” shows the value of 4. The inflammatory indexes for groups “nC_60_ EC” and “nC_60_ SC” were 14 and 18, respectively. Thus, the skin samples histological analysis showed that the nC_60_ therapy improved histological picture reducing an allergic inflammation by approximately 42 and 25 %, respectively for EC and SC applications compared with the untreated mice.

## Discussion

The investigation of fullerene C_60_ biological effects has attracted increasing attention in recent years. An important issue is that a crystalline fullerene C_60_ is practically insoluble in an aqueous medium without special processing. In this study, an aqueous solution of the fullerene was prepared by novel dialysis method [46]. Based on the spectral and elemental analyzes, we can speculate that process underlying the C_60_ solubilization can involve a formation of the C_60_ molecule complexes or their clusters with NMP followed by partial hydroxylation of nanoparticle surface that is capable of stabilizing the nC_60_ aggregates. Apparently, these aggregates are surrounded by firmly bound water envelope; the water is not removed on drying under high vacuum. We suppose, that the bond between C_60_ and NMP has definitely non-covalent character (donor–acceptor bond) as evidenced by experimental results and theoretical calculations [47]. It should be noted that in course of time (>6 months), we have observed a decrease in the heterogeneity of nanoparticles size, they have become more uniform in size (100 nm). Perhaps, it reflects the establishment of a dynamic equilibrium in a system where a process of fullerene molecules distribution from cluster to cluster takes place.

We suggest that a new method for the nC_60_ preparing as a biocompatible process is potentially very suitable for medical use. The relationship between basic physicochemical characteristics and toxic effects (in vivo) of nC_60_ practically have not been covered in scientific literature. The relationship between zeta potentials and size of functionalized C_60_ aggregates and their influence on the toxicity against bacterial cells was recently described. It was shown, that an increase of surface charge (indifferently, + or −) always leads to a decrease in the size of nanoparticles, but the toxicity is always associated with positively charged C_60_ aggregates [48]. Nanoparticles in the nC_60_ always bear a negative charge. In other studies, dimensional effects have been shown to affect the cytotoxicity of nC_60_. Authors speculate that this effect may be associated with cell-contacting surface of nanoparticles. The size of nanoparticles is negatively correlated with their toxicity [49]. The question of C_60_ water-soluble form toxicity is closely linked to the nanoparticle morphology and its surface chemistry, which is also determined by the method of its preparation. The surface of the nC_60_ particles differs from those described in the literature, in view of the presence of complexed hydrophilic component, NMP, and partial hydroxylation [46]. Our studies on safety of the nC_60_ upon acute intravenous administration to BALB/c mice demonstrated that no lethal outcomes were observed and the body weight was increased in a pattern similar to the control group (unpublished observations). Exact dose for humans will be defined at the stage of clinical trials only.

Some researchers have found that fullerene derivatives, as fullerenol and amino-fullerene, inhibit in vitro the IgE-dependent degranulation of mast cells, secretion of Th2 cytokines and prostaglandins in response to an allergen stimulation that appears to be, in part, through the cellular ROS levels inhibition [23]. Certain fullerene derivatives have been able to prevent the development of inflammation and edema in mice after administration of phorbol-myristate-acetate (PMA) [24]. The inhibition has been shown to depend on the structure of addends attached to carbon skeleton. For example, fullerene C_70_, containing 4 glycolic acid molecules, noticeably inhibited artificially induced anaphylaxis, and at the same time, fullerene with 4 attached inositol molecules showed no activity. It was shown that the fullerene material in contact with serum medium or in the cells is capable of binding to albumin and other proteins, including some enzymes. Thus, perhaps it modulates both enzymatic and signaling redox processes in the cell, but available data are very limited [25].

Earlier, based on a DTH mouse model, it was shown that the fullerene treatment significantly attenuated the footpad inflammation compared with DTH-control and switched the cytokine balance towards Th1-dominance [50]. Later we have also shown that Th1 cytokines production was significantly suppressed, however it was intriguing that Th2 cytokines IL-4 and IL-5 were also significantly suppressed by a fullerene treatment [31].

These studies were undertaken in order to evaluate the OVA-induced Th1 and Th2 cytokine secretion and to determine whether the treatment with nC_60_ can shift Th2 to Th1 response and modulate the proinflammatory cytokine production. Th2 cells produce IL-4 and IL-5 that have a prominent role in immediate-type hypersensitivity and apparently are involved in the initial stages of AD.

The level of Th1 cytokines, including IL-12 and IFN-γ was also examined. IL-12, key factor of T-cell differentiation into Th1 cells, plays a dominant role in many inflammatory diseases like AD and stimulates the IFN-γ production [51]. Thus, we demonstrated that therapeutic treatment of AD mice with nC_60_ shifts immune response from Th2 to Th1 for example changing the IgG1/IgG2a ratio as markers for Th2 and Th1 responses. We do not know the exact mechanism, but one can assume that this phenomenon is associated with the action of the nC_60_ on a redox homeostasis. ROS and other redox active molecules fulfill key functions in immunity and Th1/Th2 shift which appears to be crucially dependent on the activation of redox-sensitive signaling cascades [52].

Immune regulation and tolerance are essential functions of the immune system to prevent and limit harmful immune responses to self- and non-self-antigens. CD4/CD25 regulatory T cells (Tregs) [53] represent a unique lineage of immunoregulatory cells in both humans and animals and play a central role in the maintenance of immunologic self-tolerance and are involved in the release of anti-inflammatory cytokine IL-10 [54]. Lack of Foxp3 + CD4^+^CD25^+^ T cells leads to immune dysregulation and affected patients often have AD-like skin lesions, increased IgE levels, and enhanced Th2 responses. However, conflicting results regarding the numbers and functions of Tregs in AD have been reported. Some investigators [55] demonstrated the absence of Foxp3 + Tregs in patient’s skin that suggests a disregulation in process of inflammation. In contrast, other authors [56] showed the elevated number of circulating CD4^+^CD25^+^ Tregs with a normal suppressive function in patients with AD. However, they used the CD25 molecule expression only (without Foxp3) as a marker for Tregs.

This experiment clearly shows that the EC treatment by fullerene leads to increased expression of Foxp3 and may shed light on the mechanism of nC_60_ therapeutic effect.

The weakening of the skin barrier function in patients with mutations in filaggrin gene (FLG) probably promotes increased penetration of allergens by transdermal route. There is a direct interrelation between the AD and nonsense mutations in a filaggrin encoding gene [3]. Hence, one of possible therapeutic strategy to regulate AD is upregulating the FLG expression [57]. Recently, Otsuka et al. have screened more than 1000 compounds in a bioactive chemical library to find candidates to stimulate FLG mRNA expression and have revealed the compound JTC801 promoted the FLG mRNA and protein expression in vitro and in vivo. Potential utility of such therapy is indicated by the fact that a modest 20 % increase in filaggrin copy number leads to the 40 % reduction in AD susceptibility [25, 58].

Based on histological data and the cytokine secretion profile switch from Th2 to Th1 pattern we can conclude that fullerene nC_60_ has significant anti-allergic and anti-inflammatory activity. We observed a significant suppression of IgE and Th2 cytokines (IL-4 and IL-5) production, and with a concomitant increase of Th1 cytokines production: IL-12 (at EC application only) and IFN-γ. However, there was a difference in nC_60_ effect depended on the administration route. More intense specific IgE and Th2 cytokines suppression were observed at the EC application. In addition, this treatment significantly increased the IL-12 level compared with “AD”. Based on this facts, we can hypothesize that nC60 reduces AD inflammation by activating the IFN-γ production and Th2 response suppression [58]. These results suggest that nC_60_ might be used as an agent to suppress proinflammatory cytokine production.

Since AD is a chronic disease, apparently the barrier dysfunction is a leading primary cause of AD. Surprisingly, it turned out that the nC_60_ quite markedly increases the filaggrin expression in vivo (Fig. 5). We could not find any scientific publications on the effect of fullerene on filaggrin gene activation except the data from presentation of “Vitamin C60 BioResearch Corporation”, where C_60_/PVP complex (“Radical Sponge”) increased FLG expression in RS-treated cells control cells about a four times (http://www.novac60.com/wp-content/uploads/2013/10/VC60-Fullerene-skin-barrier-effect.pdf). However, these data once again point out the potential of fullerene C_60_ as a stimulator of the filaggrin production.

Histological analysis revealed that positive therapeutic effect was achieved both at EC and SC nC_60_-treatment, but the former was more effective. The main dermal inflammatory response component was an eosinophilic infiltration and a pronounced reduction of eosinophils number was observed in “nC_60_ EC” group. It should be noted that this result correlated with the decrease in the IL-5 concentration in the same group combined with the regenerative processes in the skin as opposed to “AD”.

Thus, in this study we have demonstrated that nC_60_ application inhibits the inflammatory process and may represent a perspective therapeutic approach to control allergic inflammation. However further studies are needed to understand the mechanism of fullerene activity.

## Conclusions

We have found that the nC_60_ inhibits significantly specific IgE production in mouse AD model. In addition, it was shown that IL-4, IL-5 levels were significantly decreased after EC and SC C60-treatment. It was observed that EC C_60_-treatment shifts immune response from Th2 to Th1, markedly increasing the production of IL-12 and IFN-γ. We have also revealed that the use of nC_60_ in EC route increases Foxp3 and FLG expression. Thus, simultaneous increase of Foxp3 and filaggrin expression leads to reduction in AD susceptibility. The histological analysis of skin samples showed that the nC_60_ therapy improved histological picture reducing an allergic inflammation via EC as well as SC applications compared with the untreated mice. We do not know exactly why EC application is more effective then SC one. The possible explanation is that EC application with nC_60_ increases the fullerene availability to the immune system due to the presence of a large amounts of immune cells in the skin with allergic inflammation (AD).

Thus, the use of nC_60_ in form of EC application is a promising alternative therapeutic approach to control allergy inflammation. Exact dose for humans will be defined at the stage of clinical trials only.

## Methods

### Reagents

Ovalbumin (OVA) (Grade V, 99 %) and l-alanine were purchased from Sigma-Aldrich (USA). N-methylpyrrolidone (NMP, 99 %) was from Panreac (Spain). Crystalline fullerene C_60_ was purchased from SES Research (99.9 %, catalog 600–9969, USA).

### nC_60_ preparation

Aqueous fullerene dispersion, nC_60_, was obtained by method described earlier [39]. Briefly, 20 mg of C_60_ were dissolved in 25 ml of N-methylpyrrolidone (magnetic stirrer) and resulting dark brown-purple solution was mixed with solution of 40 mg l-alanine in 100 ml of deionized water. The obtained dark-red transparent solution was stirred for 1 h and then subjected to exhaustive dialysis (cut off 10/50 kDa) against deionized water. Final dialysis solution was filtered through 0.45 mm nitrocellulose membrane resulting in a clear transparent solution with brownish-yellow color with concentration 120 µg/ml.

### Mice sensitization (AD model) and nC_60_ treatment

Female BALB/c mice ages 4–6 weeks were purchased from the animal nursery Filial SCBMT “Stolbovaya” (Moscow region, Russia) and kept in a pathogen-free environment with an OVA-free diet. All experimental procedures were carried out according to order no. 708 of the Ministry of Health of the Russian Federation and “Regulations on the ethical attitudes to laboratory animals NRC Institute of Immunology FMBA of Russia (Moscow, Russia)”.

EC sensitization of mice to induce a skin inflammation was carried out as described by Spergel et al. (1998). Briefly, mice were shaved with an electric razor. OVA (100 µg) in PBS (100 µl) or PBS as placebo was placed on a 1 × 1 cm^2^ patch of sterile gauze, which was then secured onto the skin with a transparent bioclusive dressing (Systagenix Wound Management Limited, United Kingdom). The patch was applied thrice over a 1-week period. An inspection at the end of each sensitization period confirmed that the patch remained in place. C_60_ treatment was carried out by subcutaneous (SC; 0.1 mg/kg) and epicutaneous nC_60_ (EC; 1 mg/kg) administrations, between OVA-applications as shown in Fig. 1.

### Antibody and cytokine assay

The anti-OVA IgE, IgG1, and IgG2a antibodies levels in sera obtained before, during, and after nC_60_-treatment were detected by ELISA (ELISA kits from BD, USA) according to the manufacturer’s protocol. OVA was used for coating the plates. Mouse anti-ovalbumin IgE mAb (AbD Serotec, UK) and biotin rat anti-mouse IgE (BD) were used for detection anti-OVA mouse IgE. These components were used to construct the calibration curve and then to analyze sera.

The spleens were taken after the last allergen application, and levels of IL-4, IL-5, and IL-12 (p40) in supernatants of OVA-stimulated splenocytes were determined by using ELISA [Duo-Set from R&D Systems (UK) and ELISA set from BD (USA)] according to the manufacturer’s protocol.

### Real-time PCR

The total RNA from OVA-stimulated mice splenocytes was extracted using the RNeasy Mini Kit (Qiagen, Courtaboeuf, France) according to the manufacturer’s instructions. The RNA concentration was determined, and cDNA was synthesized through a reverse transcription reaction (“Reverta-L”, Interlabservice, Russia). Quantitative real-time PCR analysis of mRNA expression was done by the iQ5 system (Bio-Rad, USA) and the PCR-Mix kit (Sintol, Russia).

The results are presented as mRNA expression (Foxp3, FLG). Calculations to determine the relative level of gene expression were made using the comparative C_t_ method (ΔΔC_t_) referring to the mHPRT in each sample; the results are presented as arbitrary units.

### Histological analysis

The skin specimens from patch areas were removed for histologic examination immediately after the last EC application with OVA. Skin biopsies were taken from similar body sites, fixed overnight with 10 % paraformaldehyde at 4 °C, and embedded in paraffin. Four-micrometer sections were stained with hematoxylin and eosin (H&E). The histological preparations were analyzed under a light microscope (Leica DM2000, Germany) with 50×, 100×, and 400× lenses.

### Statistical analysis

The data are shown as mean ± SE. Statistical analysis was done with the program Statistica 8.0 (StatSoft Inc., USA). The significance of the results was determined by using Student’s t test. Differences were considered significant at p < 0.05. The Quantitative RT-PCR data were calculated by using the comparative C_t_ method (ΔΔC_t_).
